# Recent Methods for the Viability Assessment of Bacterial Pathogens: Advances, Challenges, and Future Perspectives

**DOI:** 10.3390/pathogens11091057

**Published:** 2022-09-16

**Authors:** Kieu The Loan Trinh, Nae Yoon Lee

**Affiliations:** 1 Department of Industrial Environmental Engineering, Gachon University, 1342 Seongnam-daero, Sujeong-gu, Seongnam-si 13120, Korea; 2 Department of BioNano Technology, Gachon University, 1342 Seongnam-daero, Sujeong-gu, Seongnam-si 13120, Korea

**Keywords:** viability assessment, viable but nonculturable bacteria (VBNC), culturability, metabolic activity, membrane integrity

## Abstract

Viability assessment is a critical step in evaluating bacterial pathogens to determine infectious risks to public health. Based on three accepted viable criteria (culturability, metabolic activity, and membrane integrity), current viability assessments are categorized into three main strategies. The first strategy relies on the culturability of bacteria. The major limitation of this strategy is that it cannot detect viable but nonculturable (VBNC) bacteria. As the second strategy, based on the metabolic activity of bacteria, VBNC bacteria can be detected. However, VBNC bacteria sometimes can enter a dormant state that allows them to silence reproduction and metabolism; therefore, they cannot be detected based on culturability and metabolic activity. In order to overcome this drawback, viability assessments based on membrane integrity (third strategy) have been developed. However, these techniques generally require multiple steps, bulky machines, and laboratory technicians to conduct the tests, making them less attractive and popular applications. With significant advances in microfluidic technology, these limitations of current technologies for viability assessment can be improved. This review summarized and discussed the advances, challenges, and future perspectives of current methods for the viability assessment of bacterial pathogens.

## 1. Introduction

Throughout history, humanity is continuing the fight against bacterial pathogens, but it has been met with challenges, as bacterial infectious diseases are among the leading causes of mortality worldwide [1,2]. Along with the development and improvement of food processing, drinking water treatment, and sanitation, the threats of infectious diseases from the environment have been significantly reduced. However, many bacteria persist in food, water, and environmental samples even when these samples have been treated to remove the contamination [3,4]. Therefore, a method that can evaluate the viability of bacterial pathogens in food, water, and environmental samples is critical in decreasing the risks of microbial infections.

**Figure 1:**
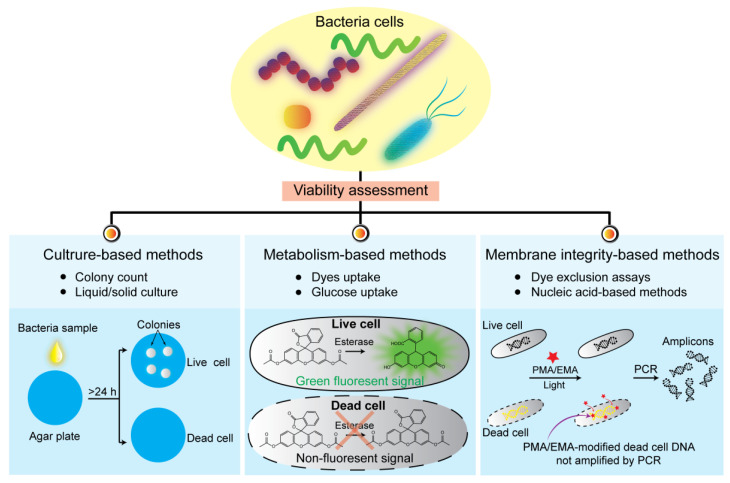
Summary of the representative methods for determining bacterial viability including culture-based methods, metabolism-based methods, and membrane integrity-based methods. PMA, propidium monoazide; EMA, ethidium monoazide; PCR, polymerase chain reaction. Red star, PMA/EMA.

To evaluate the viability of bacterial pathogens, culturability, metabolic activity, and membrane integrity are three widespread and accepted criteria [5]. The bacterial culturability can be measured by determining their ability to produce a colony—A visible mass of bacteria originating from a single mother cell when plated on an appropriate solid media. In order to form a colony, bacteria must be reproducible and metabolically active and have an intact bacterial membrane [6]. However, sometimes bacteria can enter the viable but nonculturable (VBNC) state due to unfavorable conditions, such as low temperatures, low-nutrient environments, and high antibiotic concentrations [7,8,9,10,11,12]. When bacterial pathogens enter the VBNC state, they cannot be evaluated based on culturability criteria. As an alternative strategy, VBNC bacteria can be detected by measuring their metabolic activity. Numerous studies have reported that VBNC bacteria can be evaluated by measuring the uptake of substrates, such as fluorescent dyes and glucose [13,14,15,16]. However, VBNC bacteria can enter the dormant state in which the metabolic activities of VBNC bacteria are inactive [17,18]. As a result, bacteria in the dormant state cannot be detected by measuring metabolic substrates. Therefore, another criterion for viability assessment has been introduced, relying on membrane integrity. In this approach, a dead bacterium would have a disrupted and/or broken membrane, whereas a live bacterium has an intact membrane [19,20,21]. Based on three accepted criteria (culturability, metabolic activity, and membrane integrity) for bacterial viability, various methods have been developed for evaluating bacterial viability (Figure 1). This review summarized the current methods for the viability assessment of bacterial pathogens and discussed their advances, challenges, and future perspectives.

## 2. Viability Assessments Based on Culturability

As a traditional method, the plate culture method has been widely accepted for detecting bacterial viability for >100 years [22]. This technique was first discovered by Robert Koch in 1881 for culturing, detecting, and quantifying viable bacteria [23]. A contaminated sample can be plated on an agar plate, followed by incubation for various times at various temperatures depending on the bacterial species. After incubation, viable bacteria form colonies, whereas nonviable bacteria do not [24,25]. Different bacterial types can form colonies with different shapes, sizes, and colors. Culture-dependent methods not only provide information about bacterial viability but can also be helpful in identifying bacteria [26]. However, the culture-dependent method must combine with other technologies, such as biochemical tests, Gram staining, catalase test, and sporulation test, for bacterial identification [27,28]. Quantifying viable bacteria using the culture-dependent method requires manual steps, such as spreading samples on an agar plate and counting colonies. Recently, automated instruments for spreading have been well developed and are even available in the market, such as Microstreak^®^ and commercial spiral platers [29,30]. For counting bacterial colonies, various automatic systems have been published. For example, Zhu et al. reported an automatic analysis system for counting bacterial colonies based on images captured with near-infrared light [31]. This system is convenient and cost-effective for counting colonies automatically by processing images. It takes 11–21 s to count colonies on each agar, with an average relative error of 0.2%. In another study, Molina et al. used Scan^®^ 500 (Interscience) to capture images and digitalize them to count the number of *Escherichia coli* colonies [32]. Therefore, an automated system reduces time consumption and manual steps. Although automatic systems could improve the efficiency of culture-dependent methods, the process requires 2–3 days for bacterial isolation and up to 1 week to obtain the final results of viability and quantification. Furthermore, as stated earlier, the most serious limitation of culture-dependent methods is that they cannot detect VBNC bacteria.

## 3. Viability Assessments Based on Metabolic Activities

### 3.1. Dyes Uptake Assay

**Figure 2:**
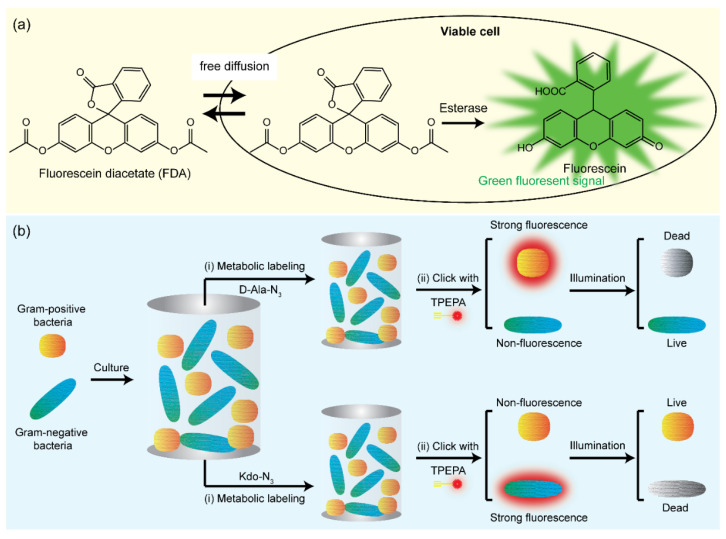
(**a**) Schematic illustration of the working principle of bacteria counting using FDA [34]. (**b**) Schematic illustration of the synthesized fluorescence turn-on TPEPA dye for discrimination and precise ablation of pathogens [42].

The metabolic activity of viable bacteria can be detected based on their uptake of dyes through bacterial membranes. When the dyes enter the bacterial membrane, they are hydrolyzed by an active enzyme system, such as esterases, lipases, and proteases, to convert nonfluorescent to detectable fluorescent signals. Fluorescein diacetate (FDA) is a common dye used for viability assessment based on the activity of nonspecific enzymes, as mentioned above, and it is a nonpolar and nonfluorescent dye. FDA has lipophilic properties because it comprises two acetate groups and is, therefore, permeable to lipid bilayer membranes of bacteria [33]. After the transportation of FDA into bacteria, it is hydrolyzed to fluorescein by nonspecific intracellular enzymes and releases measurable fluorescent signals (Figure 2a) [34]. Fluorescein is a polar molecule and, therefore, cannot move across bacterial lipid membranes. As a result, fluorescent signals are accumulated inside bacteria. The advantages of this technology are as follows: (1) FDA uptake does not require any specific transport pathway through the membranes because FDA can enter bacteria via a passive transport mechanism, and (2) extracellular FDA does not produce any background signal [33]. However, FDA carries severe disadvantages [35]. First, the quenching effect can occur when the fluorescein concentration inside bacteria is too elevated. Second, the FDA-based method is highly sensitive to pH. The acidic environment can enhance the protonation of fluorescein, which can enhance the efflux of fluorescein via passive efflux, decreasing the fluorescent signal. The product of FDA hydrolysis is acetic acid; therefore, it can decrease the intracellular pH. The pH also affects intracellular enzyme activity; each enzyme has different optimal pH conditions. Because plenty of intracellular enzymes catalyze FDA hydrolysis, optimizing the pH condition for FDA hydrolysis is difficult [36,37,38]. Nevertheless, biological metabolism-based fluorescent labeling can greatly improve bacteria labeling using the combination of a fluorescent probe and a bio-orthogonal group. Furthermore, it can be employed as an effective approach for the discrimination of peptidoglycan of gram-positive bacteria and lipopolysaccharide of gram-negative bacteria [39,40,41,42]. For example, a bacteria-metabolizable dual-functional probe TPEPy-d-Ala was developed for fluorescence turn-on imaging of bacteria based on aggregation-induced emission [39]. When metabolically bound with bacterial peptidoglycan, the mobility of the TPEPy-d-Ala probe is inhibited, resulting in clear visualization of intracellular bacteria by fluorescence signal enhancements. In another study, Wu et al. designed a bio-orthogonal fluorescence dye (TPEPA) for discriminating gram-positive (*Staphylococcus aureus*) and gram-negative (*E. coli*) bacteria by metabolic engineering (Figure 2b) [42]. Due to the different structures of bacteria morphology, this synthesized TPEPA dye could distinguish live and dead bacteria via selective imaging of metabolically decorated gram-negative bacteria with Kdo-N_3_ and gram-positive bacteria with D-Ala-N_3_ under a fluorescence microscope, respectively.

### 3.2. Glucose Uptake Assay

Glucose is a monosaccharide composed of an aldehyde group (–CHO) and six carbon atoms. Compared to other carbon sources, such as fructose, sucrose, and lactose, glucose is more abundant and can be found in most beverages. In energy metabolism, almost all organisms, including bacteria, use glucose as the main source of energy and the building block for biopolymers in all kingdoms of life [43]. Some viable bacteria would consume glucose from the environment into their cytoplasm through the membrane transport system. Once glucose is imported into the cytoplasm, it is metabolized through different pathways to become ready-to-use energy [44]. Therefore, glucose content has become one of the most important parameters for evaluating the metabolic activity of bacteria.

Methods for viability assessment based on glucose can be categorized into two main strategies: using artificial fluorescent glucose and using enzymatic assays. For the first strategy, glucose uptake can be measured using artificial fluorescent glucose, namely, 2-[N-(7-nitrobenz-2-oxa-1,3-diazol-4-yl)amino]-2-deoxy-D-glucose (2-NBDG). Only viable bacteria with active metabolisms can consume 2-NBDG via a glucose transporting system. Once 2-NBDG is incorporated into bacteria, it is decomposed as a nonfluorescent compound. Meanwhile, dead bacteria cannot degrade 2-NBDG, resulting in the remaining fluorescent signals. However, not all bacteria can consume 2-NBDG. *Vibrio mimicus* 10393, *Bacillus cereus* JCM 2152, *Plesiomonas shigelloides* NP321, *Aeromonas hydrophila* JCM 1027, and *E. coli* W539 could not take in 2-NBDG [45,46,47]. Moreover, a fluorescent spectrophotometer or a fluorescence microscope is usually required to analyze fluorescent signals that could limit their application in low-resource settings.

For the second strategy, the remaining glucose can be measured by enzymatic assays. In the presence of glucose oxidase, glucose is oxidized to form D-gluconic acid and H_2_O_2_. After that, the H_2_O_2_ level is measured by a colorimetric reaction with *o*-dianisidine under the catalyzation of peroxidase to switch *o*-dianisidine from a colorless to a colored compound [48,49,50]. Generally, enzymatic assays are expensive, and natural enzymes have low stability and are difficult to store. With the development of nanotechnology, nanozymes have been developed to address the limitations of natural enzymes. Nanozymes are nanomaterials with enzyme-like activities. Unlike natural enzymes, nanozymes are low-cost and easy to store and have high stability and tunable catalytic activities [51]. Given these advantages, glucose oxidase- and peroxidase-mimicking nanozymes for glucose assays have proven their potential for viability assessment [52,53]. In general, detecting viable bacteria using enzymatic assays, along with significant advances in nanozymes, obviously have many advantages over the fluorescent glucose-based method, including (1) the method is suitable for almost all bacteria, (2) the results can be observed by the naked eye, and (3) the method is cost-saving and portable because it does not require bulky machines, such as a fluorescence microscope or fluorescent spectrophotometer.

### 3.3. Adenosine Triphosphate (ATP) Assay

ATP Bioluminescence assay is inspired by the enzymatic reaction in fireflies, which releases detectable light by converting luciferin to oxyluciferin in the presence of ATP and luciferase [54]. ATP is an energy-carrying molecule that is essential for the metabolic activities of living organisms [55]. Therefore, ATP is widely used as a marker for viable cells, which are detected by luciferin–luciferase luminescence reactions with increasing light intensity correlating to a higher number of live cells [56,57]. The reaction can be performed within minutes and does not require heavy equipment, having assay kits with portable luminescence detectors that are already available on the market by many suppliers [57]. However, since ATP is a common energy currency for all living cells, using ATP as a marker for live bacteria might result in misinterpretation if the samples contain non-bacterial or extracellular ATP [58,59]. In addition, the ATP level can vary between bacterial species and depends on the physiological states, making direct interpretation of ATP levels to bacteria counts unreliable [60,61].

## 4. Viability Assessments Based on Membrane Integrity

As mentioned earlier, among the three criteria (culturability, metabolic activity, and membrane integrity) for viability assessments, membrane integrity is the most reliable criterion. Bacteria can enter the states allowing them to silence the reproducibility and metabolic activity; therefore, some bacteria cannot be detected using the culturability and metabolic activity criteria. However, membrane integrity is critical for bacterial function and survival [62].

### 4.1. Dye Exclusion Assays

**Figure 3:**
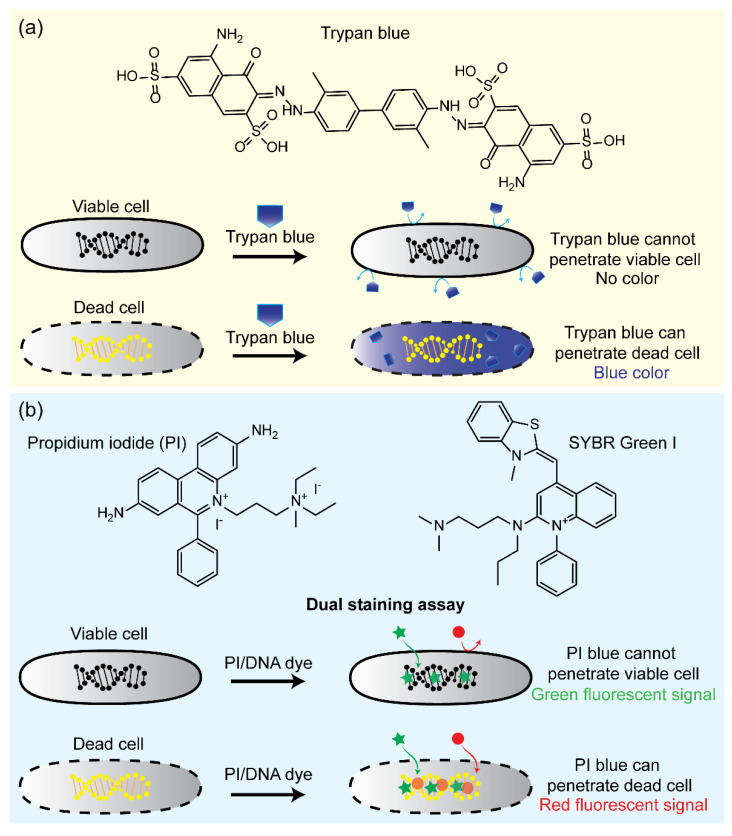
(**a**) Schematic illustration of the working principle of bacterial viability using trypan blue dye. (**b**) Schematic illustration of the working principle of bacterial viability using PI/SYBR Green I assay [78]. Green star, DNA dye; Red circle, PI.

For the viability assessment of bacteria based on membrane integrity criteria, the dye exclusion assay is one of the most common and useful techniques. The mechanism of the dye exclusion assay is based on the fact that bacteria with intact membranes are highly selective concerning the dyes that can move across their membrane, whereas a compromised membrane permits easy access. When dyes enter bacteria with damaged membranes, they interact with intracellular proteins or nucleic acids and release detectable fluorescent signals [63,64,65]. As a typical example, trypan blue is an anionic hydrophilic azo dye widely used to stain dead cells (Figure 3a). Due to its high negative charge, it is excluded from the bacteria with intact membranes. In contrast, dead bacteria can take up trypan blue because they lose membrane selectivity. After entering the bacterial cytoplasm, trypan blue interacts with cytoplasmic proteins and emits blue fluorescent signals [66,67]. The relative number of live and dead bacteria can be measured using a fluorescence microscope [68,69], light microscope [70,71,72], or flow cytometer [71,73,74] by counting unstained and stained bacteria.

As another example of dye exclusion assays, propidium iodide (PI) is also widely used for the viability assessment of bacteria, especially after the report of Boulos in 1999 [75]. Like trypan blue, PI only stains dead bacteria because it can only penetrate bacteria with compromised membranes. However, PI does not interact with intracellular proteins as trypan blue; instead, PI intercalates to DNA and RNA inside dead bacteria. The reaction with nucleic acids enhances ~30-fold the fluorescence of PI and shifts the excitation/emission maximum of PI to 535/617 nm, whereas free PI has an excitation/emission maximum of 493/636 nm [76]. The fluorescent signal can be analyzed using fluorescence microscopy, flow cytometry, or confocal laser scanning. For viability assessments, PI is usually coupled with dyes that can penetrate and stain nucleic acids of live and dead bacteria, thereby obtaining total bacteria counts. Khan et al. optimized the staining protocol and flow cytometry to detect VBNC and VC bacteria within 70 min [77]. Various fluorescent probes, such as SYTO 9, SYTO 13, SYTO 17, SYTO 40, and PI, were performed to qualify VBNC and VC *E. coli* O157:H7, *Pseudomonas aeruginosa*, *Pseudomonas syringae*, and *Salmonella enterica*. Recently, a highly sensitive approach using DNA dyes for bacterial viability was suggested by Feng et al., who used SYBR Green I and PI dyes for identifying *S. aureus, E. coli, Klebsiella pneumoniae*, *Mycobacterium tuberculosis*, and *Acinetobacter baumannii* in <30 min (Figure 3b) [78]. Another protocol was based on the dual SYTO9/PI staining assay to rapidly detect *Staphylococcus* and *P. aeruginosa*, and fluorescent signals were observed by fluorescence microscopy [76]. SYTO 9 can penetrate live and dead bacteria regardless of their membrane integrity, intercalate to DNA and RNA, and release a green fluorescent signal. Because PI exhibits a stronger affinity toward nucleic acids than SYTO 9, PI can replace SYTO 9 when both dyes are exposed to the same nucleic acid. As a result, dead bacteria are stained by PI with a red fluorescent signal, whereas a green fluorescent signal released from SYTO 9 represents live bacteria [79,80,81,82].

Although dye exclusion assays are one of the most common methods among viability assessments mentioned above, a major disadvantage of this approach is it cannot distinguish different bacterial species. In other words, dye exclusion assays can only evaluate the ratio of viable and nonviable bacteria; they cannot provide information about which bacterial species are present in the samples. This drawback may limit the application of dye exclusion assays in identifying viable pathogens.

### 4.2. Nucleic Acid-Based Methods

**Figure 4:**
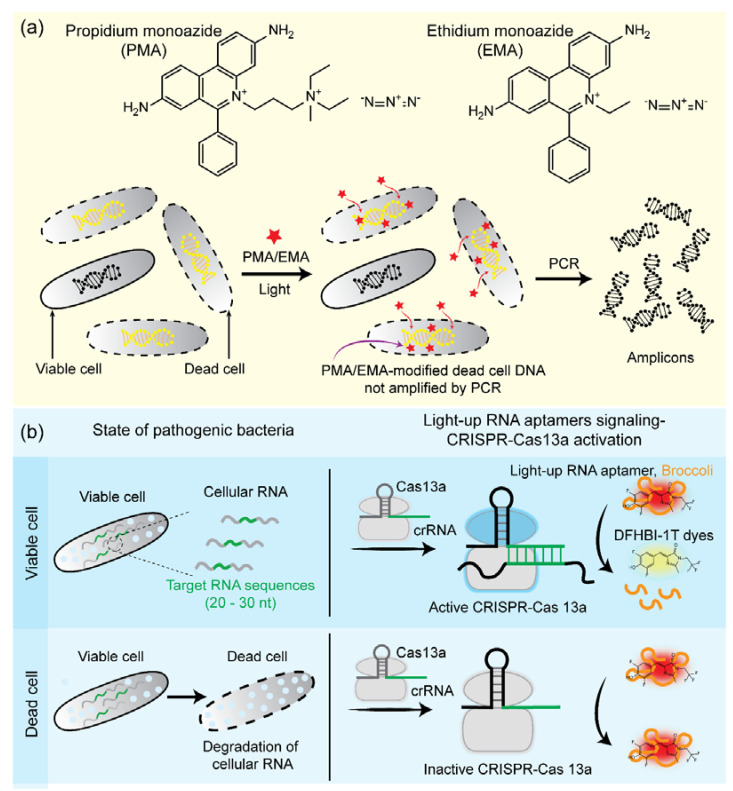
(**a**) Schematic illustration of the working principle of bacterial viability using the combination of PMA/EMA staining with polymerase chain reaction (PCR). Red star, PMA/EMA. (**b**) Schematic illustration of light−up RNA aptamer signaling−clustered regularly interspaced short palindromic repeats (CRISPR)/Cas13a for mix−and−read detection of viable pathogenic bacteria. Broccoli is a special RNA aptamer sequence that can bind and turn on specific dyes, and it was designed to serve as the signal reporter for CRISPR−Cas13a [112].

In recent years, by coupling photoreactive DNA-intercalating dyes, biomolecular techniques [e.g., polymerase chain reaction (PCR), loop-mediated isothermal amplification (LAMP), and recombinase-aided amplification (RAA)] have been widely applied for bacterial viability assays (Figure 4a) [83,84,85,86]. In this approach, DNA-intercalating dyes, such as ethidium monoazide (EMA) and propidium monoazide (PMA), are used to penetrate only nonviable bacteria with compromised membranes, whereas viable bacteria with intact membranes should pose a barrier for DNA-intercalating dyes [87,88]. Vondrakova et al. suggested that the killing methods and species-specific differences can affect EMA/PMA-quantitative PCR (qPCR) efficacies because some bacterial species are resistant to the EMA/PMA pretreatment technique [89]. In addition, to enhance the selectivity and sensitivity, a new DNA modification dye (named PMAxx, an improved version of PMA) has been developed and applied for bacterial viability assays [90,91,92]. After the penetration of dyes, they are exposed to bright visible light to stimulate the interaction between dyes and DNA [93,94]. EMA and PMA contain an azide group that can be converted into a highly active nitrene radical under exposure to bright visible light. The active nitrene radicals can bind covalently to DNA from nonviable bacteria, whereas unbound nitrene radicals are simultaneously inactivated by reacting with H_2_O in samples. The covalent bond between DNA and nitrene radical changes the DNA structure in nucleotide angle, inhibiting DNA elongation by polymerases. Nitrene radicals also reduce the solubility of DNA, enabling DNA removal by the DNA extraction process. Dye-treated samples undergo DNA extraction, followed by PCR to amplify DNA from viable bacteria. In contrast, DNA from nonviable bacteria cannot be amplified because of the covalent bond between DNA and nitrene radicals [95,96,97,98,99]. A combination of PMA dye and qPCR is the most popularly studied for the viability assay of various bacteria, such as *S. aureus*, *E. coli* O157, *P. aeruginosa*, *Lactobacillus* spp., *M. tuberculosis*, *B. cereus*, etc. [100,101,102,103,104,105,106]. For example, Li et al. introduced viability PCR with PMA and DyeTox13-qPCR methods for detecting the *inv*A gene from *Salmonella typhimurium* [107]. In this study, by optimizing the DyeTox13 assay with EMA, the PCR signal from dead cells was reduced, which helped to overcome the main limitation of the PCR approach concerning its inability to discriminate dead from live bacteria. In other words, false-positive results from dead bacteria were eliminated using this method. In 2019, Cao et al. developed real-time PCR and LAMP approaches to detect *Vibrio parahaemolyticus* in shrimp samples, which achieved the limit of detection (LOD) of ~10.5 colony-forming units (CFU)/mL [90]. Using the PMA-LAMP approach, VBNC *E. coli* O157:H7 and *S. enterica* were successfully detected and quantified in fresh produce [108]. In another study, Xu et al. proposed a modified PMAxx dye combined with RAA to detect viable *S. aureus* in milk samples, and the LOD was ~10^2^ CFU/mL [86]. Recently, apart from DNA-based methods, RNA-based methods are also being used for bacterial viability assays using RNA as an indicator [109,110,111]. Viable vancomycin-resistant *Enterococcus* was successfully discriminated against using reverse transcription LAMP for RNA amplification combined with colorimetric detection within 1 h [111]. As an alternative platform for bacterial viability, many studies tried to detect live bacteria without requiring nucleic acid extraction and amplification as usual [112,113]. Remarkably, with the advancement of clustered regularly interspaced short palindromic repeats (CRISPR)/CRISPR-associated proteins (Cas), Zhang et al. recently introduced a light-up RNA aptamer signaling-CRISPR/Cas13a principle for identifying live *B. cereus* without requiring transcription and amplification (Figure 4b) [112]. The system could detect ~10 CFU of *B. cereus* in spoiled food. Adapting the same concept, Wei et al. developed a highly specific and sensitive detection method based on the aptamer-based Cas14a1 to determine live *S. aureus* with a LOD of ~400 CFU/mL live cells [113]. Therefore, this new approach allows live bacterial detection without amplification based on Cas14a1 and a pathogenic aptamer. Although this new approach could open a new way for live bacteria with high sensitivity and specificity compared to other amplification approaches, the LOD is higher, and the total time was >150 min.

### 4.3. Microfluidic Technology for Viability Assessments

**Figure 5:**
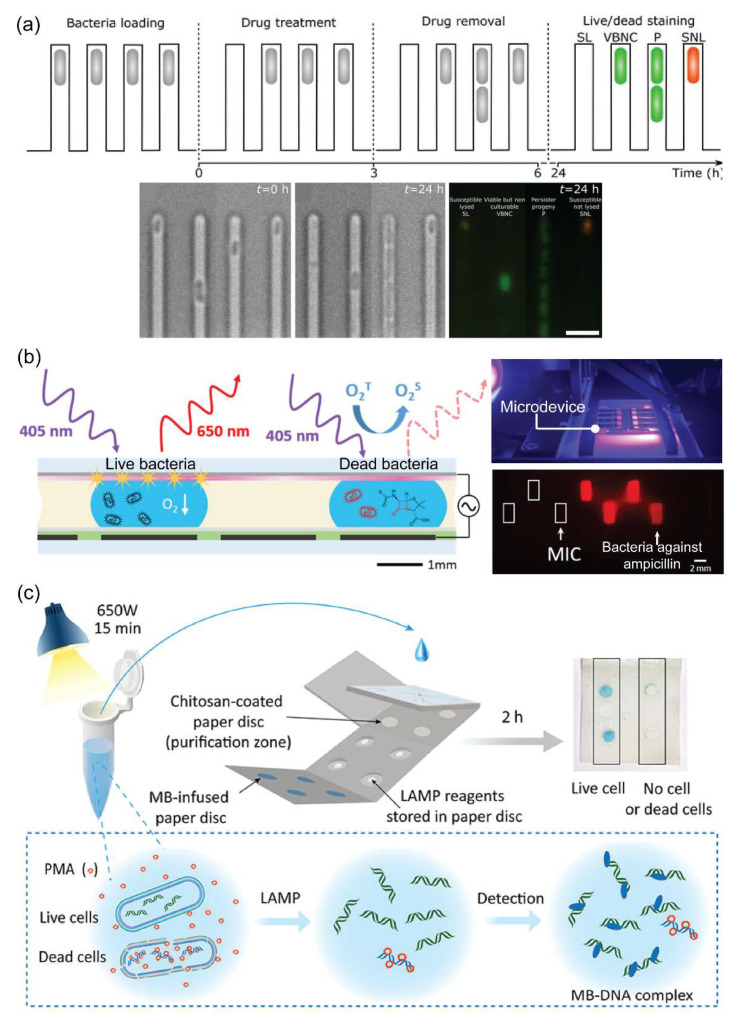
Representative microfluidic devices for the bacterial viability test. (**a**) A novel single-cell approach to study VBNC *E. coli* cells using microfluidic channels combined with time-lapse microscopy. The schematic illustrates a step-by-step procedure to distinguish VBNC cells from susceptible nonlysed (SNL), susceptible lysed (SL), and persister (P) cells. Adapted with permission from Ref. [121]. Copyright 2017, Springer Nature. (**b**) An integrated digital microfluidic chip with an oxygen sensor for an *E. coli* culture droplet applied in antimicrobial susceptibility test. The photos show a real image of a microdevice observed under ultraviolet light after a 16 h on-chip culture of *E. coli*. MIC, minimum inhibitory concentration. Adapted with permission from Ref. [122]. Copyright 2021, American Chemical Society. (**c**) A fully integrated origami microdevice for live bacterial identification based on nucleic acid analysis. MB, methylene blue; LAMP, loop-mediated isothermal amplification; PMA, propidium monoazide. Adapted with permission from Ref. [125]. Copyright 2019, American Chemical Society.

In recent years, microfluidics, also known as lab-on-a-chip, has been introduced and gained much attention from the public due to its wide-ranging applications in different fields, especially in cell biological research [114,115,116,117]. Generally, microfluidic technology is a fast-rising system that offers the integration of various processes into a single microdevice (micrometers to centimeters in size). Microdevices can offer highly efficient, sensitive, and rapid analysis with low energy consumption [118]. Due to these advantages, various viability assessments based on microfluidics have emerged for discriminating between live and dead bacterial cells [119,120]. For example, Bamford et al. introduced a combined system, including microfluidic channels and time-lapse microscopy, for observing VBNC cells before, during, and after drug treatment based on a fluorescent signal from SYTO 9 dyes (Figure 5a) [121]. Using this microdevice, a series of actions, such as culturing bacteria, treating drugs, and staining live/dead cells, could be performed simultaneously under microscopy and could capture images to investigate phenotypic or genotypic heterogeneity. In contrast, Qiu et al. fabricated a digital microfluidic device for an antimicrobial susceptibility test using an optical oxygen sensor film (Figure 5b) [122]. By measuring extracellular dissolved oxygen, this device could allow on-chip culturing and monitoring of *E. coli* growth (red fluorescent signals) with minimal sample handling and lower-volume cultures. In 2015, Chang et al. invested an integrated microfluidic system to rapidly detect live *Staphylococcus* from joint fluid as a medical application, helping with immediate medical decisions and antibiotic choices [123]. The bacterial sample was incubated with EMA and vancomycin-conjugated magnetic beads for distinguishing live bacteria and amplified 16S for bacterial typing by PCR. Using a PDMS integrated device, live bacteria were successfully detected within 30 min, and the LOD was ~10^2^ CFU per reaction. For foodborne pathogen detection, Etayash et al. used the microfluidic cantilever for the in situ detection and discrimination of *Listeria monocytogenes* and *E. coli*, and the microdevice could detect bacteria at a concentration of single-cell per microliter [124]. Briefly, a biomaterial microcantilever embeds a microfluidic channel where the internal surfaces are chemically or physically functionalized with receptors that can selectively capture bacteria. Moreover, this device also can serve as a high-throughput device for the real-time detection of bacteria and allows discrimination between intact and dead *E. coli* and their metabolic response to antibiotics based on the presence of metabolic activity. In another study, Tung et al. introduced a paper-based microfluidic device that integrated treatment and molecular biology to detect viable *E. coli* O157:H7 and *Salmonella* spp. (Figure 5c) [125]. The paper-based device employed the most advanced techniques, such as chitosan-based DNA extraction, isothermal amplification (LAMP), and colorimetric detection, for screening multiple pathogens. In another study, *E. coli* was successfully discriminated against using microfluidics based on a centrifuge platform to perform a LIVE/DEAD BacLight bacterial viability assay [126]. For environmental application, Zhu et al. reported a high-resolution three-dimensional printed microdevice for *E. coli* detection using an integrated PMA-PCR device [127]. Especially, an on-chip PMMA pretreatment was used to improve the accuracy by eliminating the need for pipetting steps. As a global public health issue, *M. tuberculosis* is a bacterium that causes serious disease, namely tuberculosis, and is slower in growth than other infectious bacteria; thus, making it difficult and challenging for early detection [128]. Recently, Wang et al. introduced an integrated microfluidic system to automatically detect live *M. tuberculosis* and distinguish dead bacteria from clinical samples [129]. In this study, using this fully integrated microdevice (including bacterial capture, PMA treatment, lysis, and PCR quantification), *M. tuberculosis* could achieve automated detection in a single chip within 90 min, and the LOD was as low as 100 CFU. From these examples, numerous microfluidic devices have been fabricated to integrate various processes required for viability assessment, such as dye treatment, DNA extraction, amplification, and detection. DNA-based analyses using a microdevice reduce the time and cost of the bacterial viability test. Therefore, the obvious advantages provided by microfluidic technology make this approach powerful for viability assessment.

## 5. Conclusions

This review summarized the current techniques for viability assessment. Based on three viable criteria (culturability, metabolic activity, and membrane integrity), current viability assessments were categorized into three main strategies (Table 1). The earliest published viability assessment is the plate culturing method, which relies on culturability. However, this technique cannot detect VBNC bacteria. In order to evaluate VBNC bacteria, the next generation for viability assessment has been developed, which relies on metabolic activity. Although VBNC bacteria reproduce poorly, they still maintain metabolic activity by the uptake of nutrients through the bacterial membrane. Dye and glucose uptake are the most common viability assessments relying on metabolic activity. Only metabolically active bacteria can uptake and convert artificial fluorescent or natural glucose and emit detectable signals. However, bacteria can enter the dormant state in which the metabolic activities of VBNC bacteria are inactive and, therefore, cannot be detected when using metabolic activity criteria. In order to overcome this limitation, dye exclusion assays relying on membrane integrity have been developed. In this approach, fluorescent dyes, such as trypan blue and PI, are used to penetrate and stain only nonviable bacteria. The major disadvantage of this approach is that it cannot distinguish different bacteria species. With the combination between dye exclusion assays and PCR, technology for viability assessment took a giant step, which allows the determination of certain viable bacteria in samples. Generally, all listed technologies require multiple processes, bulky machines, and laboratory technicians to conduct the whole process and analyze the results. Along with significant advances in microfluidic technology, almost all processes required for detecting viable bacteria have been simply integrated into a single microfluidic device. The obvious advantages of microfluidic devices, such as cost-effectiveness, high automation, and user-friendliness, make them a potential technology for viability assessment.

## Figures and Tables

**Table 1:** Advantages and disadvantages of current viability assessments.

Method	Principle	Advantages	Disadvantages	Cost Estimation	Ref.
Culture-based method	Colony morphology Counting colonies from viable bacteria	Cost effective Evaluates viability Ability to identify bacteria	Time-consuming (>24 h) Labor-intensive Inability to detect VBNC Requirement of additional methods (e.g., staining) for bacteria identification	Low cost Optical microscope (<2 K USD)	[24,25,26]
Metabolism-based method	Metabolic activity of viable bacteria Hydrolyzed dye/glucose by enzymes (e.g., esterases, and lipases proteases) Detection of fluorescent signals from dead bacteria	Fast time for detection Ability to detect VBNC Ability to discriminate between gram-negative and gram-positive bacteria	Labor-intensive Inability to identify bacteria Requirement of an instrument (e.g., fluorescence microscope, and fluorescence-based microplate readers) Requirement of dyes/artificial fluorescent glucose	High cost Fluorescence microscopy (20 K–100 K USD) Fluorescence-based microplate readers (10 K–100 K USD)	[33,42,45,46,47,130]
Membrane integrity-based method	Membrane integrity of bacteria Amplification of nuclectic acid of viable bacteria	Fast time for detection Ability to identify bacteria High sensitivity and specificity Multiplex bacteria detection	Labor-intensive Requirement of an instrument (e.g., thermal cycler, fluorescent microscope, and flow cytometry) Requirement of dyes/DNA intercalating dyes	Very high cost Flow cytometry (50 K–500 K USD) Fluorescence microscopy (20 K–100 K USD) Thermal cycler (5 K–15 K USD)	[70,71,72,73,74,97,98,99,100,101,102,103,130]

## Data Availability

Not applicable.
